# Supplementary material for: Protein structural transitions critically transform the network connectivity and viscoelasticity of RNA-binding protein condensates but RNA can prevent it
Source: Nat Commun. 2022 Sep 29;13:5717. doi: 10.1038/s41467-022-32874-0 (PMC9522849; doi:10.1038/s41467-022-32874-0)
Supplement: Supplementary file 2 — Description of Additional Supplementary Files [file 41467_2022_32874_MOESM2_ESM.pdf]

Description of Additional Supplementary Files

Supplementary Movie 1:

Primitive path analysis applied to an aged FUS-PLD condensate at $T/T_{c,FUS}$ = 0.785 revealing the topology of the underlying inter-protein β-sheet binding network which has completely percolated through the phase-separated system. The aged condensate is kinetically arrested and presents gel-like behaviour.

Supplementary Movie 2:

Primitive path analysis applied to an aged FUS-PLD condensate at $T/T_{c,FUS}$ = 0.861 revealing the topology of the underlying inter-protein β-sheet binding network which has not fully percolated. The phase-separated condensate presents liquid-like behaviour at long timescales.
